# Respiration is essential for aerobic growth of *Zymomonas mobilis* ZM4

**DOI:** 10.1128/mbio.02043-23

**Published:** 2023-11-01

**Authors:** Magdalena M. Felczak, Matthew P. Bernard, Michaela A. TerAvest

**Affiliations:** 1 Department of Biochemistry and Molecular Biology, Michigan State University, East Lansing, Michigan, USA; 2 Department of Pharmacology and Toxicology, Michigan State University, East Lansing, Michigan, USA; California Institute of Technology, Pasadena, California, USA

**Keywords:** metabolism, respiration, electron transport, biotechnology, biofuel, *Zymomonas*

## Abstract

*Zymomonas mobilis* is an alpha-proteobacterium that is a promising platform for industrial scale production of biofuels due to its efficient ethanol fermentation and low biomass generation. *Z. mobilis* is aerotolerant and encodes a complete respiratory electron transport chain, but the benefit of respiration for growth in oxic conditions has never been confirmed, despite decades of research. Growth and ethanol production of wild-type *Z. mobilis* is poor in oxic conditions indicating that it does not benefit from oxidative phosphorylation. Additionally, in previous studies, aerobic growth improved significantly when respiratory genes were disrupted (*ndh*) or acquired point mutations (*cydA* and *cydB*), even if respiration was significantly reduced by these changes. Here, we obtained clean deletions of respiratory genes *ndh* and *cydAB*, individually and in combination, and showed, for the first time, that deletion of *cydAB* completely inhibited O_2_ respiration and dramatically reduced growth in oxic conditions. Both respiration and aerobic growth were restored by expressing a heterologous, water-forming NADH oxidase, *noxE*. Oxygen can have many negative effects, including formation of reactive oxygen species (ROS) or directly inactivating oxygen sensitive enzymes. Our results suggest that the effect of molecular oxygen on enzymes had a greater negative impact on *Z. mobilis* than formation of ROS. This result shows that the main role of the electron transport chain in *Z. mobilis* is reducing the intracellular concentration of molecular oxygen, helping to explain why it is beneficial for *Z. mobilis* to use electron transport chain complexes that have little capacity to contribute to oxidative phosphorylation.

**IMPORTANCE:**

A key to producing next-generation biofuels is to engineer microbes that efficiently convert non-food materials into drop-in fuels, and to engineer microbes effectively, we must understand their metabolism thoroughly. *Zymomonas mobilis* is a bacterium that is a promising candidate biofuel producer, but its metabolism remains poorly understood, especially its metabolism when exposed to oxygen. Although *Z. mobilis* respires with oxygen, its aerobic growth is poor, and disruption of genes related to respiration counterintuitively improves aerobic growth. This unusual result has sparked decades of research and debate regarding the function of respiration in *Z. mobilis*. Here, we used a new set of mutants to determine that respiration is essential for aerobic growth and likely protects the cells from damage caused by oxygen. We conclude that the respiratory pathway of *Z. mobilis* should not be deleted from chassis strains for industrial production because this would yield a strain that is intolerant of oxygen, which is more difficult to manage in industrial settings.

## INTRODUCTION

*Zymomonas mobilis* is a facultative anaerobe of interest for biofuel production from sugars. There are also several unusual characteristics of *Z. mobilis* that make it an interesting model for testing assumptions about bacterial physiology and metabolism. For example, unlike other preferentially anaerobic organisms, *Z. mobilis* uses the Entner-Doudoroff pathway for glycolysis (one ATP per glucose) rather than the Embden-Meyerhof-Parnas pathway (two ATPs per glucose) (1). Additionally, attempts to genetically modify *Z. mobilis* have yielded evidence that this organism carries multiple copies of its genome, with some estimates as high as 50 copies per cell (2, 3). The aerobic metabolism of *Z. mobilis* is also unusual and has been the subject of debate. Although *Z. mobilis* has a documented respiratory electron transport chain (ETC), it grows more quickly and to a higher density under anoxic conditions than under oxic conditions (4, 5). The poor aerobic growth of *Z. mobilis* is surprising because in most other organisms, respiration generates more ATPs per glucose than fermentation, and therefore, facultative aerobes typically have a higher growth yield (mg biomass dry weight/mg glucose) in the presence of oxygen. The negative effect of oxygen on *Z. mobilis* has been attributed to accumulation of the toxic metabolic intermediate acetaldehyde (6, 7). While some of the acetaldehyde is converted to acetate by an acetaldehyde dehydrogenase, residual acetaldehyde is often observed in aerobic cultures of *Z. mobilis* (8). Better understanding of the respiratory chain in *Z. mobilis* will allow us to determine whether ETC genes should be deleted from chassis strains (as suggested by Kalnenieks et al.), (9) and provide insight into why seemingly harmful genes are maintained in the genome.

**Fig 1:**
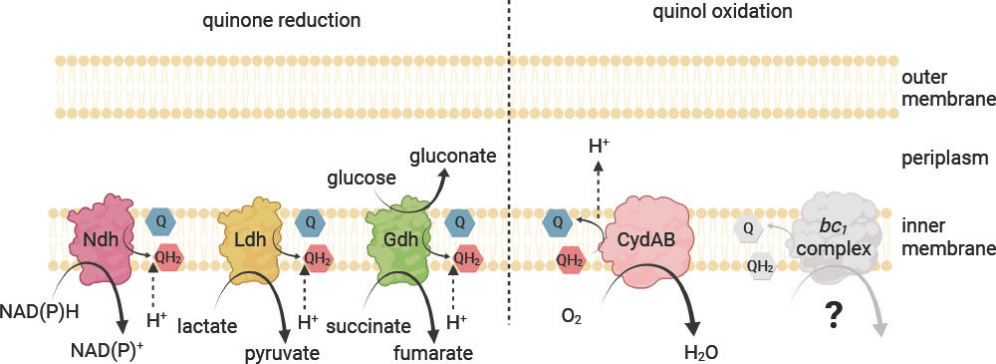
Proposed structure of the electron transport chain in *Z. mobilis*. This schematic depicts electron transport complexes encoded in *Z. mobilis*. Lactate dehydrogenase (Ldh), NAD(P)H dehydrogenase (Ndh), glucose dehydrogenase (Gdh), *bd*-type quinol oxidase (CydAB), and quinol-cytochrome *c* oxidoreductase (*bc_1_* complex). Not shown: succinate dehydrogenase. Created with BioRender.com.

The respiratory chain of *Z. mobilis* has a low predicted coupling efficiency; i.e., few protons are pumped across the inner membrane per O_2_ consumed, possibly leading to a smaller than usual benefit for using the ETC. Based on the genome sequence, *Z. mobilis* is predicted to have four dehydrogenases that donate electrons to the quinone pool: NAD(P)H dehydrogenase (Ndh, ZMO1113), quinone-linked lactate dehydrogenase (Ldh, ZMO0256), glucose dehydrogenase (Gdh, ZMO0072), and succinate dehydrogenase (Sdh, ZMO0568 and ZMO0569). A *bd*-type terminal oxidase (CydAB, ZMO1571-1572) and a *bc1* complex (quinol:cytochrome *c* oxidoreductase, ZMO0956–0958) are also encoded in the genome, but there is no known cytochrome *c* oxidase to complete the *bc_1_* pathway. Because none of the predicted ETC complexes are proton pumping, proton translocation only occurs through the oxidation and reduction of respiratory quinones at active sites on opposite sides of the membrane (Fig. 1). Compared with ETC configurations in other organisms that may translocate as many as 10 H^+^/2e^−^, the coupling efficiency of 2 H^+^/2e^−^ in *Z. mobilis* is very low and may not appreciably contribute to ATP synthesis by the *F*_o_*F*_1_ ATP synthase (10, 11). Indeed, previous work indicates that the *F*_o_*F*_1_ ATP synthase operates in the direction of ATP hydrolysis during aerobic growth by *Z. mobilis* (12).

Multiple research groups have previously shown that disruption of the gene encoding the NADH dehydrogenase in *Z. mobilis* (*ndh*) improves aerobic growth (13–16). The improved growth of respiratory mutants has raised the question of why ETC genes are conserved in the genome, since they appear to have no effect under anoxic conditions and to be deleterious under oxic conditions. Jones-Burrage et al. (13) found that while respiratory chain activity does not enhance growth, it contributes to survival in stationary phase in minimal media, as previously observed by Rutkis et al. (16). Because *ndh* mutants exhibited clear growth phenotypes and dramatically decreased oxygen consumption, they were assumed to represent the phenotype of a total lack of respiration. However, there are other entry points into the electron transport chain, including Gdh, Sdh, and a respiratory lactate dehydrogenase (ZMO0256), indicating that disrupting Ndh does not completely block the ETC (17). Further, in the absence of Ndh, the respiratory lactate dehydrogenase and the cytosolic (NAD^+^-linked) lactate dehydrogenase (ZMO1257) may form a bypass whereby NADH can be oxidized and electrons enter the respiratory chain in the absence of functional Ndh (18).

There are also unusual aspects of the latter half of the ETC. The *Z. mobilis* genome encodes a *bd* oxidase and a *bc_1_* complex but no cytochrome *c* oxidase that would be predicted to terminate the *bc_1_* branch. Sootsuwan et al. proposed that a peroxidase could terminate this branch using either hydrogen peroxide or O_2_ as the electron acceptor (19). A later study determined that while the peroxidase PerC contributed to hydrogen peroxide tolerance, it did not accept electrons from the *bc*_1_ complex or contribute significantly to the oxygen consumption rate of *Z. mobilis* (20). Strazdina et al. previously disrupted genes in the *bc*_1_ complex (*cytB*) and *bd* complex (*cydB*) by a chloramphenicol resistance marker insertion (21). These mutants had very low oxygen consumption rates initially but recovered wild type (WT)-level oxygen consumption rates after 11–12 hours of aerobic growth. This could have been due to changes in regulation or instability of the mutation.

In this study, we deleted *ndh* and *cydAB* individually and in combination and measured growth, glucose metabolism, oxygen consumption rates, and reactive oxygen species (ROS) formation in the mutant strains. In contrast to previous studies, we found that ETC flux is essential to *Z. mobilis* in oxic conditions. We rescued growth of an ETC-deficient mutant with a water-forming NADH oxidase (NoxE), which oxidizes NADH and consumes O_2_ but does not contribute to proton-motive force to drive oxidative phosphorylation. Results with NoxE indicated that oxygen removal from the environment is a key role of the ETC but not the sole benefit it provides to *Z. mobilis*.

## RESULTS

### Construction of mutant strains

To investigate the role of the electron transport chain in *Z. mobilis*, we constructed deletion mutants of the genes encoding the NADH dehydrogenase (*ndh,* ZMO1113) and the *bd* quinol oxidase (*cydAB,* ZMO1571-72) using a homologous recombination method (22). The procedure was performed in anoxic conditions as much as possible to avoid selection against the mutant strains, although exposure to oxygen was necessary at some points (see Materials and Methods). Deletion of *ndh* by this method was straightforward, but deletion of *cydAB* was more challenging. Putative mutant colonies were screened for deletion by PCR amplification of the region of interest. For the *cydAB* deletion, we observed that most colonies that produced the PCR amplicon consistent with deletion also produced an amplicon consistent with the WT sequence. Of 39 colonies screened by PCR, 6 showed the deletion band and only 1 of these also lacked the WT band (Fig. S1, isolate 5). For comparison, 8 colonies of 11 screened for *ndh* knockout showed the deletion band, and none of these contained the wild-type band with the deletion band. The *ΔcydAB* strain was also checked by Illumina re-sequencing and we found no reads aligned in the deleted region (Fig. S2). Re-sequencing indicated two point mutations across the entire genome, one in an oligosaccharide flippase family protein and one in an intergenic region of a plasmid. We also generated a Δ*cydAB*Δ*ndh* deletion strain by deleting *ndh* from the Δ*cydAB* strain.

### Growth and metabolism of mutant strains

**Fig 2:**
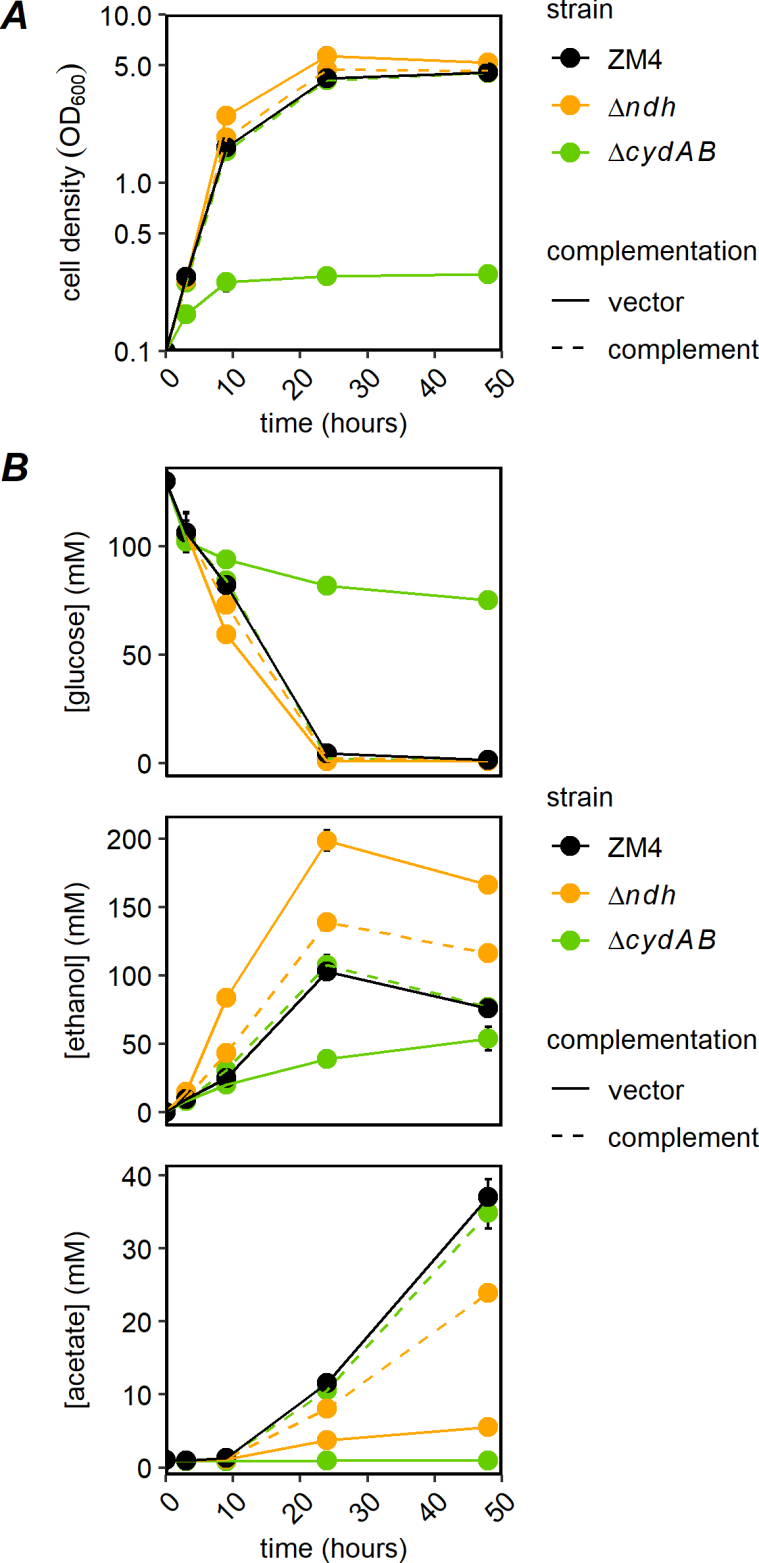
(**A**) Growth (log scale) and (**B**) substrate and product concentrations for ZM4 and Δ*ndh* and Δ*cydAB* in oxic-rich medium, with and without complementation of deleted genes. ZM4 and Δ*ndh* and Δ*cydAB* bearing the vector or respective complementing plasmids were grown in *Zymomonas* rich medium with spectinomycin in glass tubes covered with loose caps with shaking at 30°C at ambient oxygen pressure. OD_600_ was measured and samples were analyzed by high performance liquid chromatography (HPLC) as described in Materials and Methods. Points are the average of three biological replicates, and error bars are standard errors. Complement: pRL*ndh* or pRL*cydAB*.

As expected, the growth of both Δ*ndh* and Δ*cydAB* strains was indistinguishable from WT ZM4 in anoxic conditions (Fig. S3). We observed that deletion of *ndh* improved growth in oxic-rich medium (Fig. 2A, ZM4 final OD_600_ = 4.54 ± 0.06, Δ*ndh* final OD_600_ = 5.18 ± 0.15). In contrast, deletion of *cydAB* dramatically reduced growth in the same conditions (final OD_600_ = 0.29 ± 0.01). Complementation of the deletions with the corresponding genes expressed from a plasmid returned each strain to the WT phenotype, decreasing growth of Δ*ndh* and increasing growth of Δ*cydAB* (final OD_600_ = 4.63 ± 0.13 and final OD_600_ = 4.48 ± 0.14, respectively). Note that to facilitate comparisons, the non-complemented strains in Fig. 2 contain the vector, pRL814, an inducible plasmid carrying *gfp* that was used to construct both complementing plasmids. Here, we will refer to pRL814 as “vector.” This plasmid does not change the growth of WT or knockout strains when uninduced (data not shown). We observed the most complete growth complementation without isopropyl-beta-D-thiogalactoside (IPTG) induction and increasing IPTG concentration made complementation less effective (Fig. S4). We have previously observed that expression from pRL814 is leaky, so some expression of the gene of interest occurs even when inducer is not added (23).

Consistent with the improved growth, deletion of *ndh* increased glucose consumption and ethanol production and reduced acetate production compared with WT (Fig. 2B). The Δ*cydAB* strain showed low glucose consumption and ethanol and acetate production, consistent with the poor growth. In oxic conditions, both mutant strains produced ethanol more efficiently than WT (Table 1). Thus, although the Δ*cydAB* strain produced less ethanol than WT, it converted glucose to ethanol more efficiently. Again, both strains were returned to the WT phenotype (i.e., low efficiency ethanol production) by expressing the deleted gene from a plasmid. Neither mutant strain produced detectable acetaldehyde, while ZM4 accumulated 25–30 mM, possibly explaining the increase in ethanol production efficiency for the mutants (Fig. S5).

**TABLE 1:** Efficiency of ethanol production^*a*^

Strain	Plasmid	Ethanol yield (% theoretical maximum)
*ZM4*	pRL814	29 ± 2
*Δndh*	pRL814	64 ± 1
	pRL*ndh*	45 ± 3
*ΔcydAB*	pRL814	49 ± 14
	pRL*cydAB*	30 ± 3

^*a*^ Glucose and ethanol concentrations were measured by HPLC as described in Materials and Methods. Efficiency of ethanol production (%) was calculated from maximum theoretical yield produced from glucose used after 48 hours (2-mol ethanol/mol glucose).

**Fig 3:**
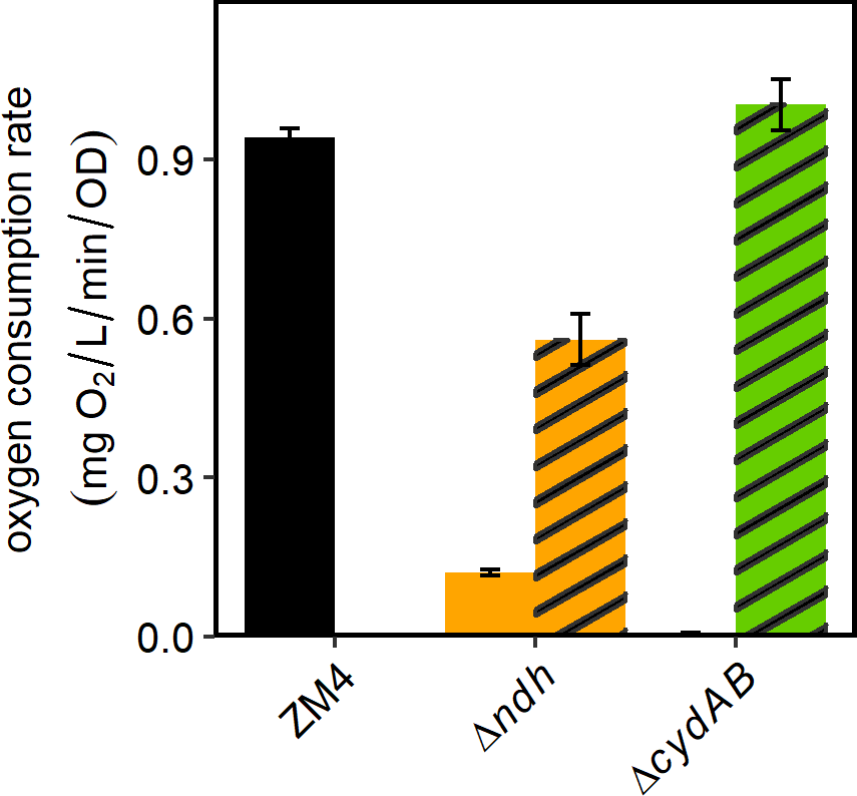
Oxygen consumption by *Z. mobilis* ZM4 and Δ*ndh* and Δ*cydAB* mutants with and without complementation. Overnight cultures were diluted in fresh *Zymomonas* rich medium with spectinomycin to an OD_600_ appropriate for oxygen measurement; ZM4/vector and Δ*ndh* and Δ*cydAB* strains with complementing plasmids were diluted to OD_600_ = 0.2, and Δ*ndh* and Δ*cydAB* bearing the vector to OD_600_ = 1.0. 200 µL from each dilution were loaded onto an OxoPlate in triplicate, and oxygen consumption was measured as described in Materials and Methods. Bar graphs are average of three biological and three technical replicates, and error bars are standard errors. Striped bars denote strains with complementing plasmids. Note that there is only one column for ZM4 with no plasmid. The bar for Δ*cydAB* is too small to be seen on this scale.

We measured the oxygen consumption rate of each strain using microplates that contained optical sensors integrated into the bottom of the wells to facilitate high-throughput monitoring (OxoPlates). Oxygen partial pressure (pO_2_) is measured as changes in fluorescence of the optical sensor. Cultures grown anaerobically overnight were diluted in fresh aerobic medium to an OD_600_ appropriate for measuring pO_2_ over time (see Materials and Methods). Cultures were aerated for 30 min by shaking in a plate reader; this time should be sufficient to induce any ETC genes that are upregulated by oxygen exposure (24). We found that ZM4 consumed oxygen at a rate of 0.941 ± 0.017 mg/L/min, while Δ*ndh* consumed oxygen at 13% of the WT rate and Δ*cydAB* consumed oxygen at 0.6% of the WT rate (Fig. 3). Complementing Δ*ndh* with *ndh* returned the oxygen consumption rate to 60% of the WT rate, and complementation of Δ*cydAB* with *cydAB* returned oxygen consumption to 106% of the WT rate (representative dissolved oxygen profiles in Fig. S6).

**Fig 4:**
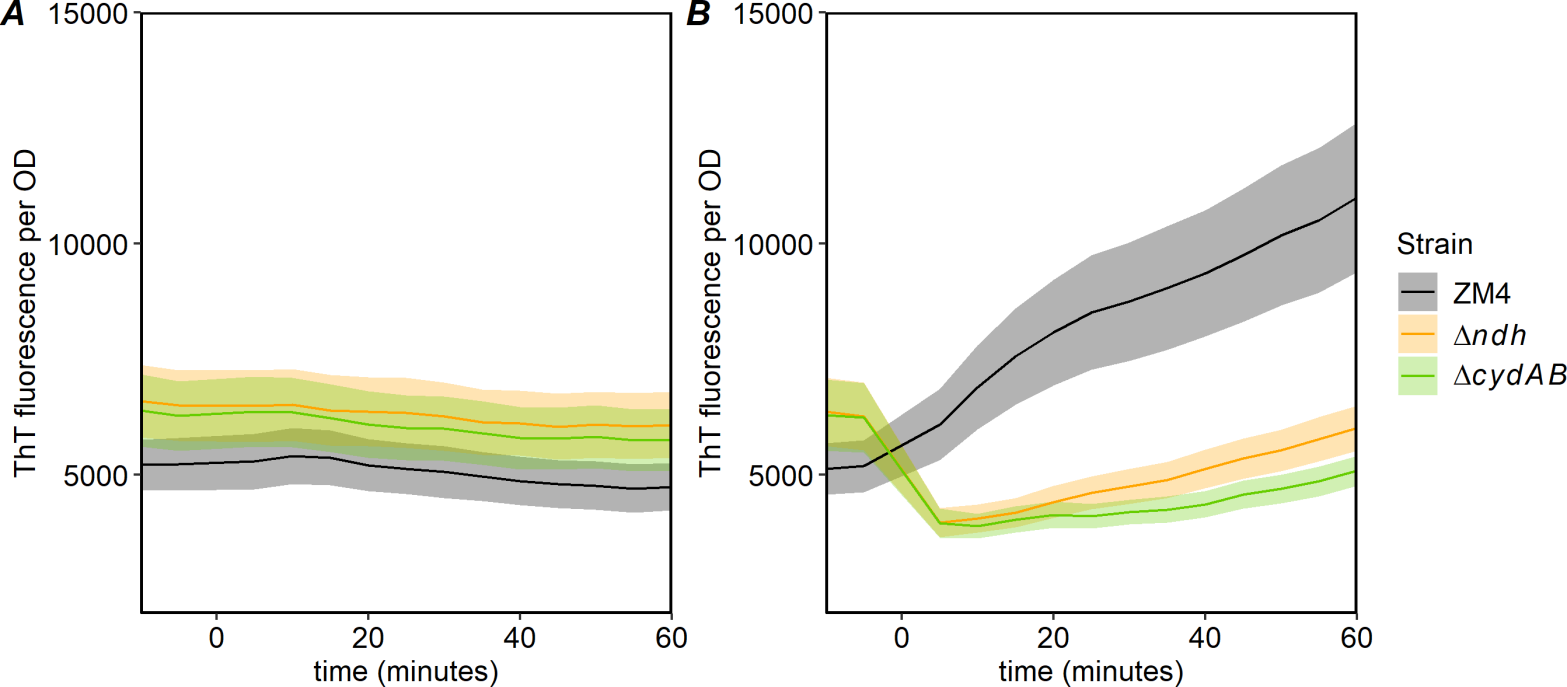
Thioflavin T (ThT) fluorescence in ZM4 and Δ*ndh* and Δ*cydAB* strains after exposure to oxygen without (**A**) or with (**B**) 2% glucose addition. Strains were grown in *Zymomonas* rich medium in anoxic conditions overnight. Cultures were pelleted by centrifugation, washed once in phosphate-buffered saline (PBS), and resuspended in PBS at final OD_600_ of 0.5. ThT was added to 10 µM, and 0.2 mL of each culture was loaded on 96-well microplate. The plate was incubated at 30°C with shaking in the plate reader, and OD_6oo_ and fluorescence (460/528 nm) were measured for 2 hours. After this time, glucose was added and measurement continued for an additional 2 hours. *X*-axis shows 10 min before and 1 hour after addition of glucose, which was set at 0. The curves are average of three biological and three technical repeats, and the shadowed area represents the standard error.

We also assessed the ability of ZM4 and the mutants to generate proton-motive force (PMF) using a cationic voltage indicator dye, thioflavin T (ThT). This dye freely diffuses into bacterial cells, and its fluorescence intensity increases as PMF increases (25). We observed that when cells were provided with glucose (Fig. 4B), ThT fluorescence rapidly increased in ZM4 (6012 ± 2319 AU/hour). Both mutant strains showed a very different response to glucose, with an initial decrease in fluorescence, followed by a slower increase (2,646 ± 513 and 1,449 ± 159 AU/hour for Δ*ndh* and Δ*cydAB*, respectively). All strains showed low, stable fluorescence in the absence of glucose (Fig. 4A).

### Analysis of a Δ*cydAB*Δ*ndh* double mutant

**Fig 5:**
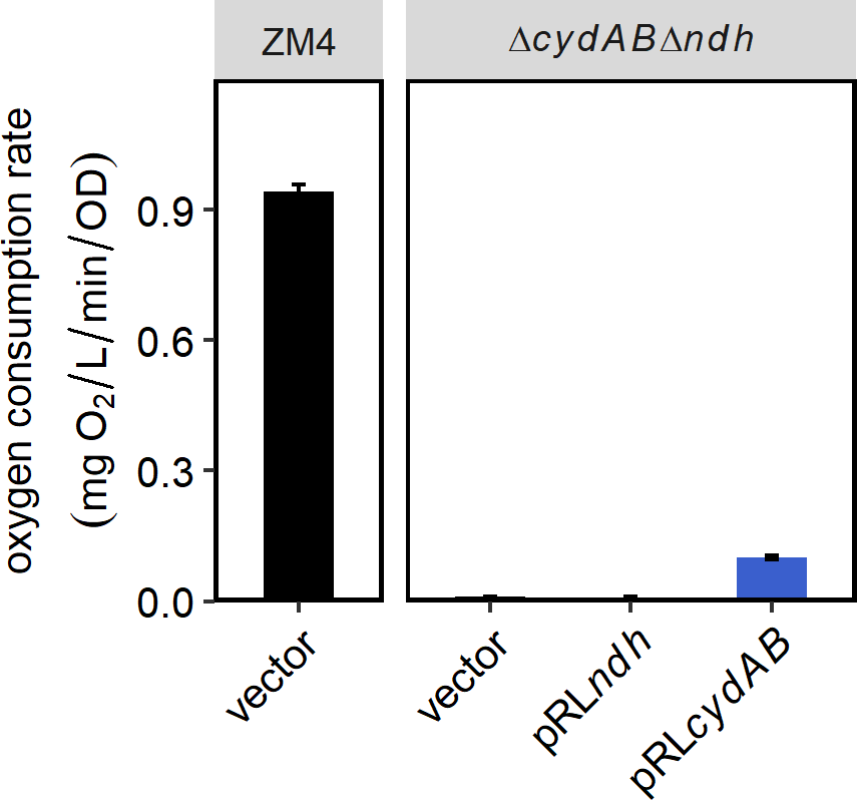
Oxygen consumption by Δ*cydAB*Δ*ndh* with complementing plasmids. Strains were grown as described in Fig. 3. ZM4/vector was diluted to OD_600_ = 0.2, while the double mutant strains bearing the vector or a complementing plasmid were diluted to OD_600_ = 1.0. O_2_ uptake rate was calculated as described in Materials and Methods and in Fig. 3. Bar graphs are the average of three biological and three technical replicates, and error bars are standard errors.

We also generated a double mutant lacking both *ndh* and *cydAB*. As expected, the oxygen uptake rate of the double mutant was negligible, as in the Δ*cydAB* strain. Complementing with pRL*ndh* did not improve the oxygen consumption, and complementing with pRL*cydAB* increased it to a level similar to the Δ*ndh* strain (Fig. 5, representative profiles in Fig. S7). As in previous figures, strains containing the vector were used as the negative control for complementation.

**Fig 6:**
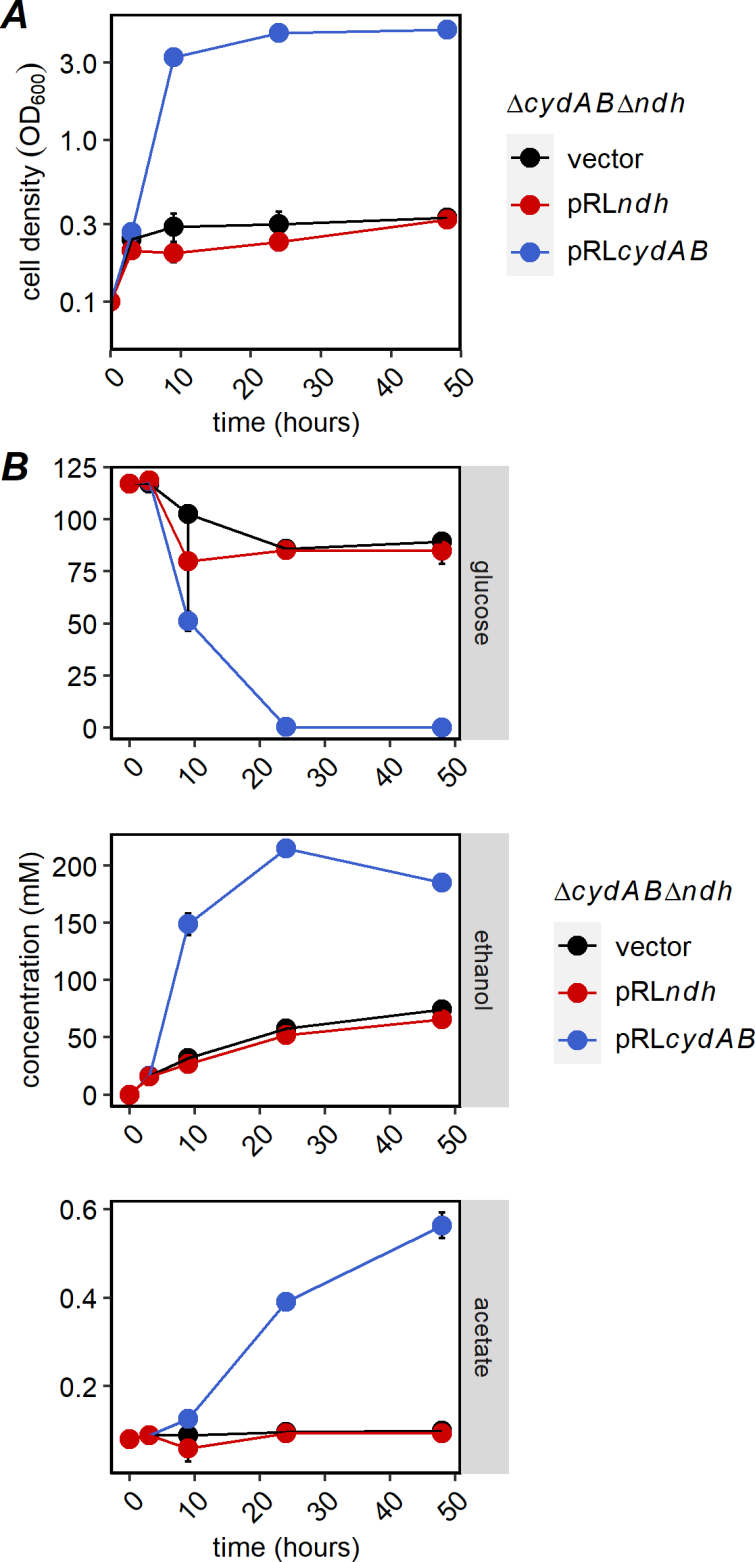
(**A**) Growth and (**B**) HPLC analysis of Δ*cydAB*Δ*ndh* with complementation. Δ*cydAB*Δ*ndh* strain bearing the vector or one of the complementing plasmids was grown in *Zymomonas* rich medium with spectinomycin as described in Fig. 2. OD_600_ was measured at times indicated to monitor growth, and supernatants were analyzed by HPLC as described in Fig. 2. Points are average of three biological replicates, and error bars are standard errors.

We observed that the double mutant had the same phenotype as Δ*cydAB*, growing very poorly in oxic conditions (Fig. 6A). Similar to the observation for oxygen consumption, complementation with *cydAB* rescued growth, while complementation with *ndh* had no effect on growth. Glucose consumption, ethanol production, and acetate production for the double mutant and complemented strains were all consistent with the growth, essentially as in the Δ*cydAB* strain (Fig. 6B). This result confirms that Ndh and CydAB are operating in the same metabolic pathway (ETC).

### Growth rescue by a water-forming NADH oxidase

**Fig 7:**
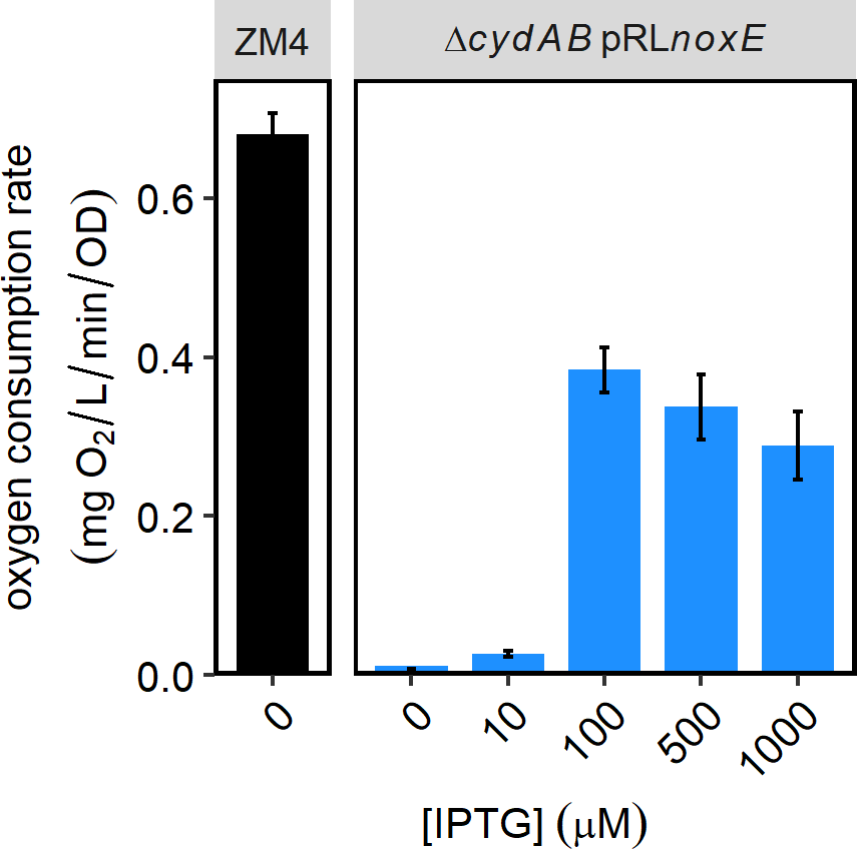
Oxygen consumption with heterologous expression of *Lactobacillus brevis noxE*. Δ*cydAB*/pRL*noxE* strain was grown in *Zymomonas* rich defined medium containing spectinomycin and indicated concentrations of IPTG, at 30°C in an anaerobic chamber overnight. ZM4/vector was grown without IPTG. Oxygen partial pressure was measured as described in Materials and Methods and in Fig. 3. Bar graphs show the average oxygen uptake rate after normalization. *n* ≥ 6 for Δ*cydAB*/pRL*noxE* and *n* = 3 for ZM4/vector.

**Fig 8:**
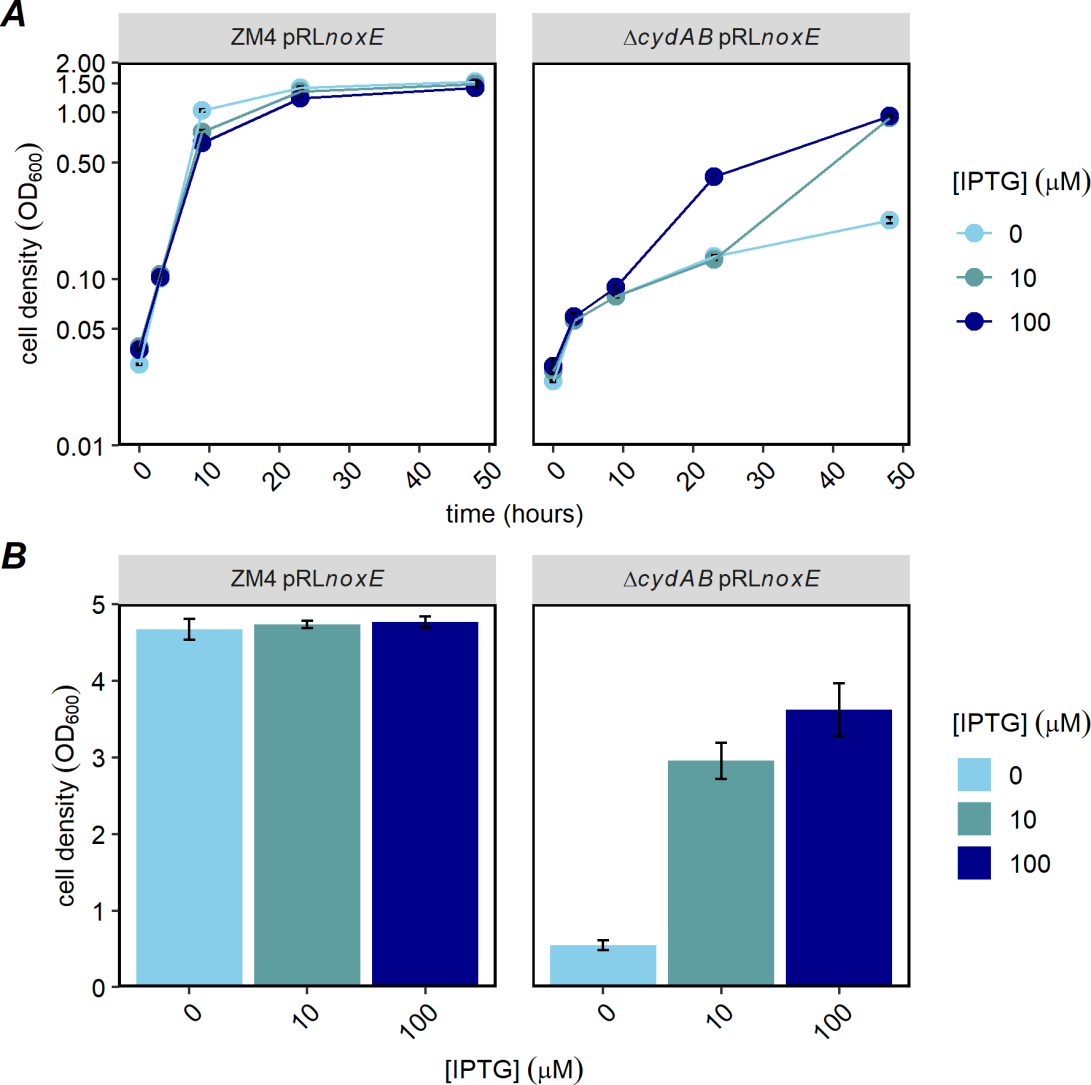
Growth of Δ*cydAB* with heterologous expression of *Lactobacillus brevis noxE*. ZM4 and Δ*cydAB* strains, bearing the vector or pRL*noxE*, were grown in *Zymomonas* rich defined medium, supplemented with spectinomycin and IPTG at the indicated concentrations. Two hundred microliters from each culture at the indicated IPTG level was loaded on a 96-well clear microplate in triplicate. Cells were grown on a plate shaker in 30°C at ambient oxygen pressure for 48 hours. Growth was monitored by measuring absorbance at 600 nm in a plate reader. After 48 hours, cultures from three wells were combined and the final OD_600_ was measured by a spectrophotometer with a 1-cm path length. (**A**) Growth curve from a representative experiment measured by plate reader; average of three technical replicates. (**B**) OD_600_ after 48 hours of incubation; average from three independent plates, with three technical replicates each. Error bars are standard errors.

CydAB may be important to *Z. mobilis* for different reasons, including reoxidation of quinols in the inner membrane, removal of O_2_ from the environment, or contribution to PMF generation for ATP synthesis or other processes. To determine whether oxygen consumption was the key role of CydAB, we recombinantly expressed NoxE from *Lactobacillus brevis*, a water-forming NADH oxidase that oxidizes NADH and reduces O_2_ to H_2_O in the cytoplasm (26, 27). NoxE consumes oxygen without contributing to PMF formation. When *noxE* was expressed in *Z. mobilis* Δ*cydAB*, we observed IPTG-dependent oxygen consumption, indicating that functional NoxE was produced (Fig. 7; Fig. S8). Maximal oxygen consumption was observed with induction with 100-µM IPTG. At this induction level, oxygen consumption of the Δ*cydAB* strain producing NoxE was 56% of the WT rate. We also measured growth at a range of IPTG concentrations and observed that NoxE expression moderately improved growth of Δ*cydAB* (Fig. 8).

### Intracellular reactive oxygen species

**Fig 9:**
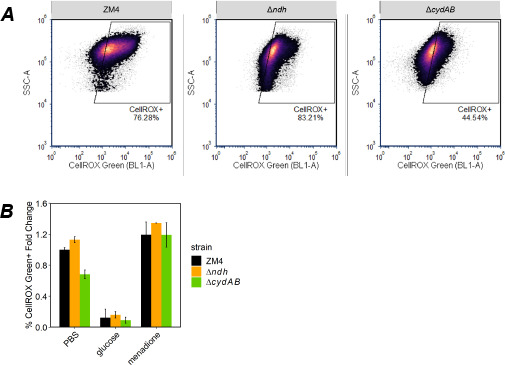
Assessment of ROS in ZM4, Δ*ndh*, *and* Δ*cydAB* strains. ZM4, Δ*ndh*, and Δ*cydAB* strains were incubated with PBS alone, PBS with glucose, or PBS with menadione. ROS formation was evaluated by assessing CellROX Green fluorescence in singlet, live (Sytox Red^ꟷ^) bacteria using flow cytometry. Representative density plots in panel (**A)** show the percentage of CellROX Green^+^ bacteria for incubation with PBS alone. The relative fold changes compared to the average percentage of CellROX green^+^ cells in ZM4/PBS samples are shown in panel (**B)**. Fold change was averaged from three to four biological replicates for each strain in up to two separate experiments. Error bars are 95% confidence intervals.

To better understand the growth defect of Δ*cydAB*, we assessed formation of ROS in the WT and mutants using a ROS sensing dye, CellROX Green. For this experiment, cells were grown in rich medium in anoxic conditions overnight. Cultures were washed in PBS and diluted to OD_600_ = 0.1 in PBS with glucose. We did not observe increased CellROX Green fluorescence in the Δ*cydAB* mutant, suggesting that the growth defect is not due to increased ROS formation. To confirm that the dye could detect ROS in all strains, we used PBS with menadione (a redox-cycling ROS generator) as a positive control. All strains showed high CellROX Green fluorescence in the presence of menadione (Fig. 9B). We also compared ROS formation for all strains in PBS without glucose to observe the effect of respiratory activity. In ZM4 and Δ*ndh*, we observed that more cells had high CellROX fluorescence without glucose than with glucose, suggesting that oxygen consumption was protective against ROS. Glucose also decreased the number of cells with high CellROX fluorescence in Δ*cydAB*, suggesting that other ROS protection mechanisms such as the respiratory peroxidase were active when glucose was present (20).

## DISCUSSION

As previously observed by other groups, we found that deletion of *ndh* from the *Z. mobilis* genome resulted in enhanced growth in oxic conditions. However, when we deleted *cydAB* from the genome, we observed a severe growth defect in oxic conditions. These results suggest that contrary to the previous hypotheses that respiration is harmful to *Z. mobilis* growth, the electron transport chain is essential to aerobic growth. We speculate that all previous respiratory deficient conditions, whether induced by a mutation or inhibitor, likely still had significant respiratory activity, resulting in the lack of a growth defect in previous studies. In the case of Ndh mutation, Strazdina et al. found that removing Ndh alone is insufficient to block respiratory activity because the NAD^+^-linked and quinone-linked lactate dehydrogenases create a bypass that allows entry of electrons into the ETC (18). Our oxygen consumption results corroborate their findings, indicating that electrons can still pass through the ETC in the absence of Ndh. The respiration deficient mutants isolated by Hayashi et al. included some large deletions or stop codons in *ndh* but only point mutations in *cydA* and *cydB*, indicating that all these mutants likely had residual respiratory activity. The point mutations in *cydA* and *cydB* were missense mutations leading to single amino acid substitutions in each case. Similar to Ndh disruption, cyanide addition to *Z. mobilis* cultures reduces the respiratory rate but does not block the ETC completely and leads to enhanced growth in oxic conditions (6). Our results show that the residual respiratory activity in all of these cases was essential to aerobic growth.

Strazdina et al. previously generated a *cydAB* mutant via insertion of a chloramphenicol resistance cassette but found that the mutant retained respiratory capacity (21). They suggested that an alternative pathway involving the *bc1* complex could compensate for the lack of the *bd* oxidase and thus respiration would still occur if only one of the pathways is removed. Our results show that only the CydAB pathway is active in ZM4 under these conditions, leaving the possible role of the *bc1* complex unknown. In Strazdina et al., growth data for the *cydAB* mutant strain were not presented, so it is difficult to compare our strain with theirs, although we suspect that, due to chromosomal polyploidy of *Zymomonas* (2, 3), they may have had a mixture of the disrupted and WT sequences. Other groups have also observed that intended gene deletions in *Z. mobilis* actually function as knockdowns rather than knockouts because each cell carries multiple genome copies, and in some cases not all copies are disrupted (2). This was observed during whole genome transposon mutagenesis of *Z. mobilis* ZM4, where many essential genes received transposon insertions, but the insertion strains were heterozygous (28). Insertions were detected in *cydA* and *cydB* in the transposon library, but they had strong fitness defects under all oxic conditions, suggesting that they were heterozygous (29). A strong fitness defect and heterozygous insertion was observed for known essential genes, including *rpoB* and *ftsZ*. The polyploidy of *Z. mobilis* likely led to the hybrid PCR results we observed during construction of Δ*cydAB*, but PCR indicated that we obtained a colony with all copies deleted (Fig. S1). Further, genome sequencing did not detect any reads in the Δ*cydAB* open reading frame, indicating that we generated a true deletion mutant (Fig. S2).

Comparing the growth and oxygen consumption rates of WT, Δ*ndh*, and Δ*cydAB*, it appears that even the low level of oxygen consumption of Δ*ndh* is very important to growth in aerobic conditions, although additional flux is not helpful. Although the ETC appears to be suboptimally regulated in laboratory conditions, we found that it is necessary for oxic growth. It remains puzzling why *ndh* has been conserved in the *Z. mobilis* genome when it seems deletion has no effect or has improved growth in the conditions tested in our study and others. Jones-Burrage et al. found that Ndh contributes to survival during stationary phase in oxic minimal medium, suggesting that there may be an important role under environmental conditions (13). Future work to simultaneously delete multiple respiratory dehydrogenases may shed light on the specific role of Ndh.

Previous work suggested that aerobic respiration is harmful to *Z. mobilis* because of acetaldehyde formation (4) and that *ndh* mutants grow better than WT because of reduced acetaldehyde concentrations. However, we observed that the Δ*cydAB* strain grew very poorly despite producing no acetaldehyde (Fig. S5). This indicates that acetaldehyde toxicity alone is insufficient to explain the poor aerobic growth of *Z. mobilis* or the growth enhancement of Δ*ndh* strains. To test whether oxygen itself is the cause of poor growth in Δ*cydAB*, we expressed a heterologous water-forming NADH oxidase, NoxE. We found that NoxE improved the final culture density to ~75% of the WT level, indicating that reducing O_2_ concentration in the culture is one of the key functions of the ETC.

Oxygen can have a range of negative effects on cells, including formation of ROS and inactivation of oxygen-sensitive enzymes. We performed intracellular ROS measurements using flow cytometry and a ROS-sensing dye to explore which aspect of oxygen is harmful to *Z. mobilis*. We found that intracellular ROS was lower in ZM4 with glucose than in starved ZM4, suggesting that respiration has a protective effect on WT, likely by reducing the oxygen concentration in the cell. Similarly, Δ*cydAB* has lower ROS levels with glucose than without. Although this strain could not have protected itself from ROS by oxygen consumption, we speculate that electrons were still fed to the cytochrome *c* peroxidase, leading to the protective effect of glucose (30). Overall, the Δ*cydAB* strain exhibited lower intracellular ROS than WT with or without glucose, suggesting that while respiration protects against ROS, CydAB is also a significant source of ROS in ZM4. Based on these results, it appears more likely that NoxE benefitted Δ*cydAB* not by protection from ROS but by protection of oxygen-sensitive enzymes from O_2_. Previous multi-omic analysis of oxygen exposure in *Z. mobilis* indicated that iron sulfur cluster enzymes were quickly inactivated by O_2_, leading to major disruptions in central metabolism (24). By expressing the electron transport chain, *Z. mobilis* can quickly remove oxygen from the environment and begin repairing iron sulfur cluster enzymes to return to metabolic homeostasis (within minutes in typical culturing conditions [in a culture tube at OD_600_ = 1.0, Fig. S9]).

Although our results show that oxygen consumption is likely the main role of the ETC, there was no condition under which NoxE fully rescued growth, suggesting that the ETC may also contribute other benefits, possibly including formation of PMF. Although the oxygen consumption rate of the NoxE producing strains was lower than WT, the oxygen consumption for Δ*ndh* was also lower, and this strain grew better than WT. Therefore, slower oxygen consumption cannot fully explain why NoxE did not fully rescue the *cydAB* deletion. Measurements using a membrane voltage indicator dye suggest that the ETC does contribute to PMF generation. The difference in dye fluorescence with and without electron donor (glucose) showed the same trend as fluorescence with and without an electron acceptor in the well-studied respirer *Shewanella oneidensis*, indicating that the dye is functioning as a reporter of PMF generation by the ETC (25). Our results confirm previous work showing that *Z. mobilis* ETC components are capable of generating PMF (31). While previous work indicated that PMF generated by the ETC could power ATP synthesis in vesicles or starved cells, it remains unclear whether PMF drives ATP synthesis during aerobic growth in this organism (31).

Although respiration is generally considered a source of ATP via oxidative phosphorylation, our results support previous findings that energy conservation is not the only or even the major role of the electron transport chain in *Z. mobilis*. Evidence that oxygen consumption is the main role of the ETC in *Z. mobilis* helps to explain why complexes with low coupling efficiency are used. If proton translocation by the ETC is not needed, complexes with low coupling efficiency may be used without any growth penalty. At the same time, use of less efficient ETC components can be advantageous because these tend to be smaller and simpler than their efficient counterparts and therefore incur less metabolic cost to produce. For example, the proton-translocating NADH dehydrogenase in *E. coli* consists of 14 subunits, while the uncoupling version has only one. Therefore, by using a low efficiency electron transport chain, *Z. mobilis* can gain the benefits of oxygen consumption with the minimum metabolic cost. This has also been observed in *Azotobacter vinelandii*, which uses a low efficiency electron transport chain to reduce intracellular oxygen levels to protect its nitrogenase from oxygen (32). Low efficiency electron transport complexes are found in a wide variety of bacteria, and future work will further expand our understanding of a likely wide range of functions they can contribute to beyond oxidative phosphorylation.

## MATERIALS AND METHODS

### Media and chemicals

*Escherichia coli* strains were grown in Luria-Bertani media (Miller, Acumedia). *Zymomonas* rich medium (ZRMG) contains 1% yeast extract, 2% D-glucose and 15-mM KH_2_PO_4_. *Zymomonas* rich defined medium (ZRDM) contains 40-mM potassium phosphate buffer, pH 6.2, 2% glucose, 0.05% NaCl, 0.1% (NH4)_2_SO_4_, 0.2-g/L MgSO4 × 7H_2_O, 0.025 g/L of Na_2_MoO_4_ × 2H_2_O, 0.01 g/L of CaCl_2_ × 2 H_2_O, 0.0025-g/L FeSO_4_ × 7 H_2_O, 0.001-g/L calcium pantothenate, 1× Teknova EZ supplement, and 1× Teknova ACGU solution. 2,6-Diamino pimelic acid (DAP) was added to 0.3 mM when indicated. Spectinomycin and chloramphenicol were used at 100 or 70 µg/mL, respectively, for *Zymomonas* or at 50 or 35 µg/mL, respectively for *E. coli*. IPTG (Sigma) was added as indicated. Restriction enzymes, T4 ligase, Q5 High-Fidelity Polymerase, and HiFi DNA Assembly Master Mix were from New England Biolabs. Oligonucleotides and gBlocks were from IDT.

### Bacterial strains and plasmids

Bacterial strains and plasmids are listed in Table 2. WM6026 [*lacIq rrnB3* Δ*lacZ*4787 *hsdR*514Δ*araBAD*567 Δ*rhaBAD568* rph-1 attλ::pAE12 (ΔoriR6K-*cat*:: Frt5), *ΔendA*::Frt *uidA* (ΔMluI)::*pir attHK*::pJK1006 (ΔoriR6K-*cat*::Frt5; *trfA*::Frt) Δ*dapA*::Frt] and broad host plasmid pRL814 bearing GFP and spectinomycin resistance gene (*aadA1*) were from Dr. Patricia Kiley and Dr. Robert Landick (University of Wisconsin, Madison). pRL814 was used as the cloning vector to generate complementation plasmids.

**TABLE 2:** Bacterial strains and plasmids

Name	Species	Relevant genotype	Reference/source
Strains			
*Mach 1*	*E. coli* K-12	W Δ*recA1398 endA1 fhuA* Φ80Δ(*lac*)M15 *Δ(lac)X74 hsdR* (r_K_*^–^*m_K_*^+^*)	ThermoFisher Scientific
*WM6026*	*E. coli* W	Pir+Δ*dap*	(33)
*ATCC 31821(ZM4*)	*Z. mobilis*	Wild type	Type Culture Collection
*ZM4ΔcydAB*	*Z. mobilis*	ΔZMO1571-72 (*cydAcydB*)	This work
*ZM4Δndh*	*Z. mobilis*	ΔZMO1113 (*ndh*)	This work
*ZM4ΔcydABΔndh*	*Z. mobilis*	ΔZMO1571-72ΔZMO1113	This work

Name	Insert	Gene source	Reference/source
Plasmids			
*pRL814*	*gfp*	*Aequorea victoria*	(34)
*pRLcydAB*	*cydAcydB*	*Zymomonas mobilis*	This work
*pRLndh*	*ndh*	*Zymomonas mobilis*	This work
*pRLnoxE*	*noxE*	*Lactobacillus brevis*	This work
*pPK15534*	*gfp*	*Aequorea victoria*	(19)
*pPK15534Δndh*	Flanks to *ndh*	*Zymomonas mobilis*	This work
*pPK15534ΔcydAB*	Flanks to *cydAB*	*Zymomonas mobilis*	This work

ZM4 deletion mutants were constructed as described before (18) using oligonucleotides listed in Table 3. Briefly, chromosomal 500-bp regions directly upstream and downstream of a target gene (flanks) were amplified from ZM4 using Q5 polymerase; primers are listed in Table 3. Both fragments were inserted into a SpeI site of non-replicating plasmid pPK15534 (GFP, CmR) by Gibson assembly (22). The reaction mixture was transformed into chemically competent *E. coli* Mach 1. Sequences of the plasmids, isolated from chloramphenicol-resistant *E. coli* Mach 1 transformants, were confirmed by Sanger sequencing. pPK15534 bearing sequences flanking a target gene was introduced to ZM4 by conjugation using WM6026 (DAP−) as a donor strain as described in Felczak *et al* (35). Selection for colonies with the plasmid integrated into chromosome was for Cm resistance and DAP independence. One colony of a ZM4-CmR conjugant was used to inoculate ZRMG without the antibiotic, and culture was grown for at least 10 generations. 10^4^ CFUs were spread on 100 ZRMG plates without chloramphenicol and left for 48 hours to grow. Colonies were screened for loss of GFP fluorescence using a blue light illuminator. Conjugation to ZM4 and resolution of primary integrants to obtain ZM4Δ*ndh* by recombination were performed in aerobic conditions. To obtain ZM4Δ*cydAB* and Δ*cydA*BΔ*ndh*, conjugation and the following steps were performed in an anaerobic chamber. In this case, plates containing single colonies were left in 4°C outside of the chamber for 24 hours for maturation of fluorescence before screening for non-fluorescent colonies. Colony PCR from primers flanking a target gene was used to confirm the gene deletion in non-fluorescent colonies. The double mutant, Δ*cydA*BΔ*ndh*, was constructed by conjugating pPK15534 bearing regions flanking *ndh* to ZM4Δ*cydA*B strain followed by selection as for single knockout strain.

**TABLE 3:** Oligonucleotides

Name	Sequence	Description
MF ndh KO upstream F	tcatgtttgacagcttatcaAACATAATCAAGACAAAAGAAAG	F,^*a*^ amplifies 500 bp upstream of *ndh*
MF ndh KO upstream R	ttgaaacctctattctcttc	R,^*a*^ amplifies 500 bp upstream of *ndh*
MF ndh KO downstream F	gaagagaatagaggtttcaaTATTTGACGCAGAAGACTTTTG	F, amplifies 500 bp downstream of *ndh*
MF ndh KO downstream R	caaggcaagaccgagcgctaATTCCTGTCTTAACCAATATC	R, amplifies 500 bp downstream of *ndh*
Up cydA F	tcatgtttgacagcttatcagagacctaagcctcttttc	F, amplifies 500 bp upstream of *cydA*
Up cydA R	tggtgtacgcatgcaaattgtattcggg	R, amplifies 500 bp upstream of *cydA*
Dwn cydB F	caatttgcatgcgtacaccatttattgc	F, amplifies 500 bp downstream of *cydB*
Dwn cydB R	caaggcaagaccgagcgctagataaatcaggacagaaagaac	R, amplifies 500 bp downstream of *cydB*
NdeI ndh F	gctacatatgtcgaagaatggtagacc	F, amplifies *ndh* for cloning to PRL814 by ligation
Bam HI ndh R	gcatggatcctcagtataatttgactttagggcg	R, amplifies *ndh* for cloning to PRL814 by ligation
MF_13	gagatatacatatggtaccagatgcgaccg	F, amplifies *cydAB* with overlaps to pRL814
MF_14	atcaagcttagtgatacccgtcactgctg	R, amplifies *cydAB* with overlaps to pRL814
MF_15	gggtatcactaagcttgatatcgaattcctgcagc	F, amplifies pRL814 with overlaps to *cydAB*
MF_16	atctggtaccatatgtatatctccttcttaaagttaaactaattctagatgtg	R, amplifies pRL814 with overlaps to *cydAB*
MF_48	tgtgactttcattgtatatctccttcttaaagttaaactaattctagatgtgt	R, amplifies pRL814 for cloning *noxE* from *L. brevis*
MF_49	taacgcttaagcttgatatcgaattcctgcagc	F, amplifies pRL814 for cloning *noxE* from *L. brevis*

^*a*^ F, forward primer; R, reverse primer.

Plasmids pRL*ndh*, pRL*cydAB*, and pRL*noxE* were constructed by introducing the indicated gene into pRL814 to replace *gfp*. All primers used in cloning are listed in Table 2. Specifically, *ndh* (ZMO1113) was amplified from ZM4 genomic DNA using primers which added NdeI and BamHI restriction sites at 5′ and 3′ ends, respectively. The fragment was then digested with the above enzymes and introduced to NdeI/BamHI digested pRL814 by ligation. *cydAB* (ZMO1571-1572) was amplified from ZM4 gDNA using primers MF_13 and MF_14 and introduced to pRL814 amplified with primers MF_15 and MF_16 by Gibson assembly. A gBlock (IDTDNA) was used for cloning of *noxE* from *Lactobacillus brevis* (AF536177.1) to pRL814. ggagatatacat and gcttgatatcga overhangs were added at the 5′ and 3′ termini, respectively, of *noxE* gene sequence. The fragment was cloned by Gibson assembly to pRL814 amplified using primers MF_48 and MF_49. All reaction mixtures were routinely transformed to *E. coli* Mach 1, and colony PCR was used to confirm insertion of the gene into pRL814. Finally, recombined pRL814 plasmids were sequenced by the Sanger method to confirm the correctness of the construct.

### DNA extraction and sequencing

Genomic DNA from a ZM4Δ*cydAB* isolate was extracted with DNeasy UltraClean Microbial Kit (Qiagen) and was further prepared for sequencing using NEBNext Ultra FS II kit (NEB) with the miniaturized protocol (36). Prepared next generation sequencing (NGS) libraries were sequenced using Illumina NovaSeq 6000. Sample sequences were compared to reference ZM4 (ATCC31821) (NZ_CP023715.1) with breseq (37) using the analysis pipeline available at https://github.com/mikewolfe/Bacterial_reseq.

### Bacterial growth

Strains were grown in indicated media in an anaerobic chamber overnight. Spectinomycin was added when indicated. In the morning, cultures were diluted to OD_600_ = 0.1 in 5 mL of the same media. For aerobic growth, bacteria were grown in glass tubes covered loosely with plastic caps. For anaerobic growth, cultures were grown in Hungate tubes. In this case, after dilution of overnight cultures with fresh, anaerobic media, the tubes were closed with rubber stoppers and secured with aluminum crimps in anaerobic chamber (gas composition ~1% H_2_, balance N_2_). Aerobic and anaerobic cultures were then incubated with shaking at 275 rpm at 30°C outside of the chamber. Samples were taken periodically for cell density and HPLC analysis.

### Rescue of Δ*cydAB* growth from pRL*noxE*

Growth rescue with pRL*noxE* was performed in ZRDM because we observed growth inhibition of ZM4 after expression of pRL*noxE* in the standard ZRMG medium. Δ*cydAB* or ZM4 bearing the vector or the *noxE* plasmid were grown in ZRDM supplemented with spectinomycin, overnight, in an anaerobic chamber. In the morning, cultures were diluted to OD_600_ = 0.1 in fresh aerobic ZRDM with spectinomycin. IPTG was added to final concentrations as indicated. Wells of 96-well, clear, flat-bottomed microplates (Greiner) were filled with 200 µL of culture in triplicate. Bacteria were grown aerobically in a 30°C incubator with shaking at 500 rpm using a portable shaker (IKA MS three digital) for 48 hours. Absorbance at 600 nm was measured periodically by plate reader (Synergy H1 BioTek). At the end of the experiment, cultures from technical replicates were combined, and OD_600_ was measured by a spectrophotometer with a 1-cm path length.

### HPLC analysis

Samples were taken at the times indicated and stored in −20°C until used. Just before HPLC analysis, samples were thawed and centrifuged at maximum speed in a benchtop microcentrifuge for 10 min, and clear supernatants were used for analysis. HPLC chromatography was performed on a Shimadzu 20A HPLC equipped with an Aminex HPX-87H, 300.0 × 7.8-mm column, at 50°C. Metabolites were eluted with 5-mM sulfuric acid at 0.6 mL/min, and detection was by refraction index based on the standards run alongside.

### Oxygen uptake measurement

#### Optical sensor microplate

Bacteria were grown overnight in ZRMG or ZRDM at 30°C in an anaerobic chamber. Cultures were diluted in fresh, aerobic medium to the initial OD_600_ indicated in the experiments. The dilution factor was determined experimentally to adjust for O_2_ uptake rate characteristic for each strain that allowed for near 100% air saturation after 30 min of shaking in a plate reader. Wells of 96-well, flat-bottomed OxoPlate (OP96C, Precision Sensing) were filled with 200 µL of each culture in triplicate. Control wells were filled with 400 µL of oxygen-free calibration solution (Cal 0%) or 200 µL of 100% air-saturated calibration solution (Cal 100%), in quadruplicate, as recommended by the manufacturer. The wells containing Cal 0% were covered with adhesive foil. Cal 0% was prepared by dissolving 0.15 g of Na_2_SO_3_ in 15-mL water in a 15-mL, tightly closed VWR vial. Cal 100% was obtained by vigorous vortexing of 20 mL of H_2_O in a 50-mL VWR conical tube for 2 min and subsequent gentle moving of liquid for 1 min to reduce air oversaturation. The vial was closed and both standards were used immediately. The plate was covered with the lid and was shaken in a plate reader in aerobic conditions for 30 min at 30°C to stabilize the temperature and to allow for expression of oxygen-inducible genes. After this time, shaking was stopped and reference (540/590) and indicator (540/650) fluorescence were measured every 3.2 min for 1 hour. Oxygen partial pressure as percentage of air saturation (pO_2_) was calculated from the formula pO_2_ = 100 × (*k*0 / IR − 1)/(*k*0 / *k*100 − 1), where IR = fluorescence indicator/fluorescence reference (Fi/Fr) of unknown sample; *k*0 = Fi/Fr of Cal 0%; and *k*100 = Fi/Fr of Cal 100%. The final value of O_2_ concentration in mg/L was obtained by multiplying pO_2_ by a conversion factor of 0.091. O_2_ consumption rate was calculated from the linear part of the slope and normalized to OD_600_ = 1.

#### Manual optical sensor

Cultures were grown in ZRMG, at 30°C in anoxic conditions, overnight. After diluting in fresh ZRMG to OD_600_ = 1.0, 40 mL was aerated in 250-mL flasks by vigorous shaking at 30°C for 30 min. After this time, 30 mL of culture was immediately transferred into a 50-mL VWR tube, and dissolved oxygen was measured every 30 seconds for 10 min, with a pre-equilibrated optical probe (InLab OptiOx Optical Dissolved Oxygen Sensor, Mettler Toledo).

### Thioflavin T fluorescence

ZM4, Δ*ndh*, and Δ*cydAB* were grown in 3 mL of ZRMG in anoxic conditions overnight. Cells were pelleted by centrifugation, and resuspended in an equal volume of PBS. The centrifugation step was repeated, and pellets were resuspended in 1 mL of PBS. Cells were diluted to OD_600_ = 0.5 in PBS, and thioflavin T was added to the final concentration of 10 µM. Six wells of a 96-well microplate (clear/black Greiner 655090) were filled with 0.2 mL of each bacterial suspension, and ThT fluorescence was measured by microplate reader for 2 hours at excitation/emission 460/528 nm, respectively (Synergy H1, BioTek). Optical density (*A*_600_) was measured at the same time. After this time, glucose was added to three out of six wells to 2%, and fluorescence was measured for an additional 2 hours.

### Flow cytometric analysis of ROS

Strains were grown in ZRMG in an anaerobic chamber overnight. Cultures were centrifuged at 14,000 rpm for 1 min at room temperature, resuspended in equal volume of PBS and diluted to OD_600_ = 0.1 in PBS with 0.5-µM CellROX Green (Invitrogen) ROS detection reagent. Glucose was added to the final concentration of 2%, and menadione was added to a concentration of 100 µM from a 10-mM stock solution in dimethyl sulfoxide (DMSO), as indicated in the experiments. All samples were incubated at 30°C for 30 min and subsequently kept on ice before being subjected to flow cytometric analysis. Sytox Red (Invitrogen) viability dye at 10-nM concentration was added just prior to acquisition. CellROX Green (BL1-A) and Sytox Red (RL1-A) fluorescence was assessed on an Attune CytPix (ThermoFisher Scientific) flow cytometer in the MSU Flow Cytometry Core Facility. Post-acquisition analysis was performed using FCS Express (v.7, De Novo Software). ROS was assessed based on CellROX Green fluorescence in bacteria gated on a FSC-A/SSC-A cellular population, singlets, and live cells (Sytox Red^ꟷ^). Gating placement for the percentage of CellROX Green^+^ bacteria was determined based on a fluorescence minus one control (Sytox Red stained only). The fold change in CellROX Green^+^ percentages were calculated with respect to the average percentage CellROX Green^+^ cells ZM4 for each sample (% CellROX Green^+^ / average % of CellROX Green^+^ in PBS-treated ZM4 cells for each experiment = relative fold change).

## SUPPLEMENTAL MATERIAL

Supplemental figures mbio.02043-23-s0001.docx Figures S1 to S9.

ASM does not own the copyrights to Supplemental Material that may be linked to, or accessed through, an article. The authors have granted ASM a non-exclusive, world-wide license to publish the Supplemental Material files. Please contact the corresponding author directly for reuse.
