# Supplementary material for: Respiration is essential for aerobic growth of Zymomonas mobilis ZM4
Source: mBio. 2023 Nov 1;14(6):e02043-23. doi: 10.1128/mbio.02043-23 (PMC10746213; doi:10.1128/mbio.02043-23)
Supplement: Supplemental figures — Figures S1 to S9. [file mbio.02043-23-s0001.docx]

Supplementary information for “**Respiration is essential for aerobic growth of *Zymomonas mobilis* ZM4”**

Magdalena M. Felczak^1^, Matthew P. Bernard^2^, and Michaela A. TerAvest^1^

^1^Department of Biochemistry and Molecular Biology, Michigan State University, East Lansing, MI, USA

^2^Department of Pharmacology & Toxicology, Michigan State University, East Lansing, MI, USA


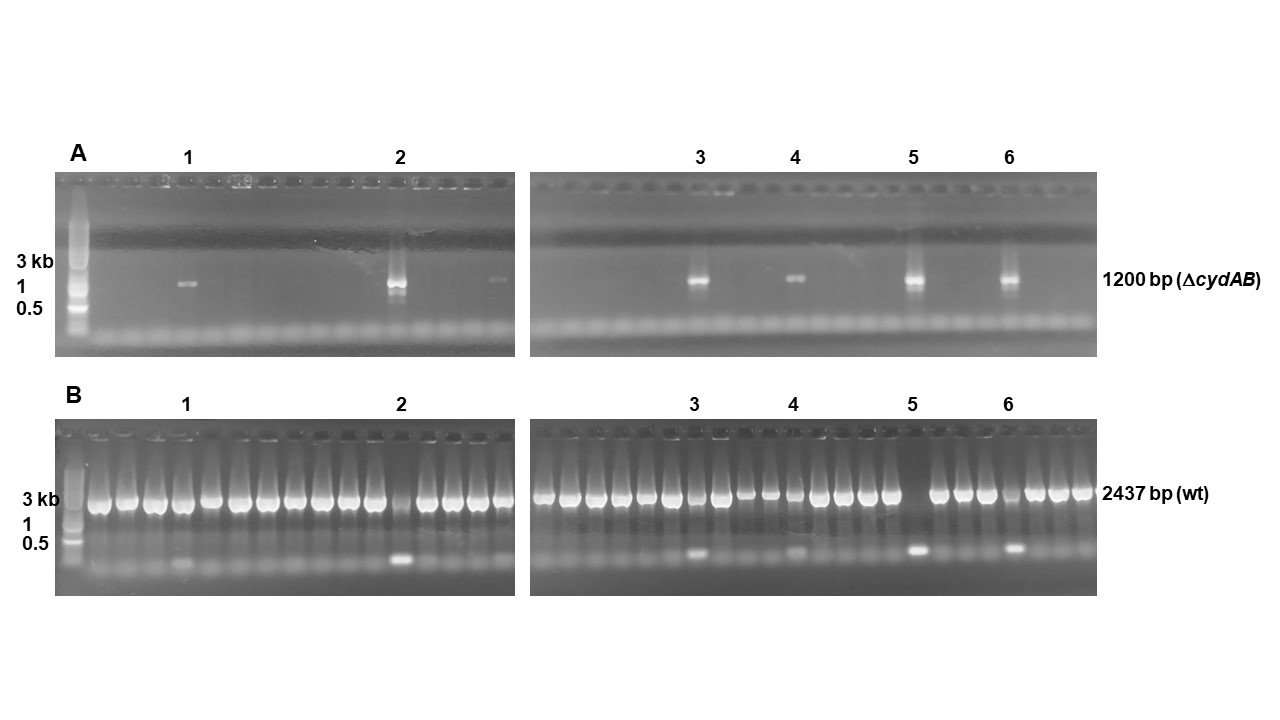


**Figure S1. PCR screening of putative Δ*cydAB* mutants.** Non-fluorescent colonies obtained after plasmid resolution from ZM4::pPK15534Δ*cydAB* primary integrants (see Materials and Methods) were colony PCR from primers upstream and downstream of the *cydAB operon* (**A**), or from primers annealing inside *cydAB* (**B**). 3 μl of PCR reaction was loaded on 0.7% agarose gel. Numbers to the right indicate expected size of a PCR product. Numbers 1-6 at the top of each gel indicate isolates that generated PCR products expected for deletion of *cydAB*. Lanes from the left: 1kb plus ladder, colony PCR 1-39

**Figure S2. Read coverage for the *cydAB* region in the Δ*cydAB* strain.** Genomic DNA from ZM4Δ*cydAB* isolate was purified with DNeasy UltraClean Microbial Kit (Qiagen). Whole genome sequencing was by Illumina. Breseq analysis was performed using ZM4 (ATCC31821) genome sequence as reference (NZ_CP023715.1). Coordinates in reference genome between 1605182- 1607642 correspond to *cydAcydB* operon. Upper tract is read coverage across entire ZM4 genome (NZ_CP023715.1) where blue trace is number of reads aligned. Lower tract is zoomed in on cydAB region. Figure made with Geneious version 2021.0 created by Biomatters.

**Figure S3. Anaerobic growth of Δ*ndh* and Δ*cydAB* mutants in rich medium.** ZM4, Δ*ndh* and Δ*cydAB* strains were grown in anoxic conditions in ZRMG, overnight. Cultures were diluted to OD_600_ = 0.1 in fresh anoxic medium in Hungate tubes and closed with rubber stoppers and secured with crimps in anaerobic chamber. Cultures were grown outside of anaerobic chamber for 24 hours. At times indicated samples were removed with a syringe and OD_600_ was measured to monitor growth. Each point is an average of three biological repeats and error bars are standard errors.

**Figure S4. Growth of Δ*ndh* and Δ*cydAB* mutants with complementing plasmids at variable IPTG.** ZM4/pRL814, Δ*ndh*/pRL*ndh* and Δ*cydAB*/pRL*cydAB* were grown in anoxic conditions in ZRMG supplemented with spectinomycin, overnight. Cultures were diluted to OD_600_ = 0.1 in fresh medium and IPTG was added to final concentrations as indicated. Cultures were grown in glass tubes with loose caps with shaking for 48 hours. OD_600_ was measured to monitor growth. Each point is an average of three biological repeats and error bars are standard errors.

**Figure S5. Acetaldehyde synthesis during oxic growth of Δ*ndh* and Δ*cydAB* mutants in rich medium.** ZM4, Δ*ndh* and Δ*cydAB* strains were grown in oxic conditions in ZRMG, overnight. Cultures were diluted to OD_600_ = 0.1 in fresh oxic medium and grown in glass tubes with loose caps with shaking at ambient oxygen level. At times indicated, samples were removed for growth and subsequent analysis by HPLC. Acetaldehyde concentration in centrifuged supernatants were calculated from standards. Each point is an average of three biological repeats and error bars are standard errors.


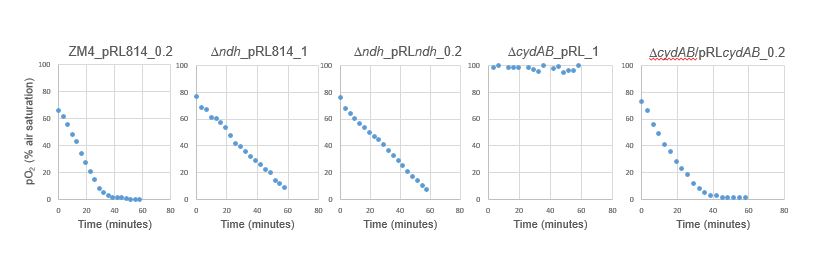


**Figure S6. Oxygen consumption profiles in ZM4 and Δ*cydAB* and Δ*ndh* with and without complementation.** Strains were grown in rich medium (ZRMG) with spectinomycin at 30^o^C in an anaerobic chamber, overnight. Cultures were diluted in fresh ZRMG with spectinomycin to OD_600_ appropriate for oxygen measurement (see text); the OD_600_ after dilution (1.0 or 0.2) is indicated in chart titles. 200 μl from each dilution was loaded onto an Oxoplate in triplicate. Plates were incubated with shaking at 30^o^C in a plate reader for 30 minutes. After this time, shaking was stopped and fluorescence was measured every three minutes for 60 minutes. Oxygen partial pressure (pO_2_) as “% air saturation” was calculated from two-point calibration of the Oxoplate, as described in “Materials and Methods”. Each graph shows oxygen consumption in one well, representative for the indicated strain.

**
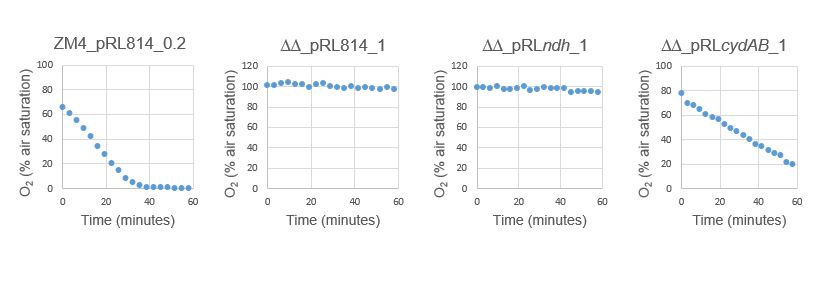
**

**Figure S7. Oxygen consumption profiles of Δ*cydAB*Δ*ndh* with complementing plasmids.** Strains were grown as described in Figure S4. ZM4/pRL814 was diluted to OD_600_ = 0.2 while the double mutant strains bearing pRL814 or a complementing plasmid were diluted to OD_600_ = 1.0. Oxygen partial pressure was measured as described in “Materials and Methods” and in Figure S4. Each graph shows oxygen consumption in one well, representative for the indicated strain.


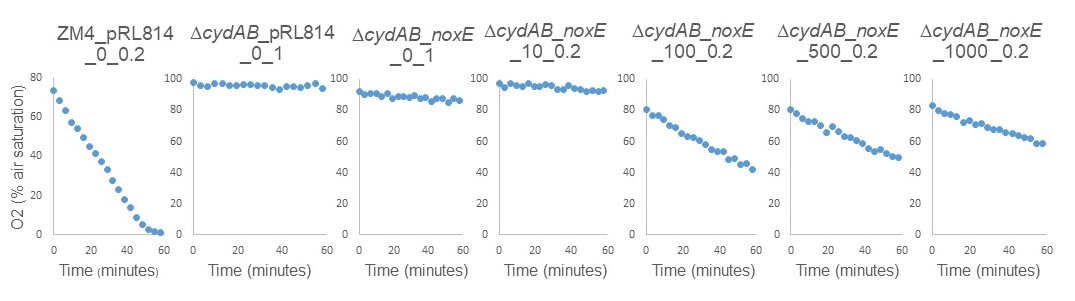


**Figure S8. Oxygen consumption profiles of ZM4/pRL*noxE* and Δ*cydAB/pRLnoxE* at different levels of induction.** Δ*cydAB*/pRL*noxE* strain was grown in rich defined medium (ZRDM) containing spectinomycin and indicated concentrations of IPTG (0-1000 μM), at 30^o^C in an anaerobic chamber, overnight. ZM4 and Δ*cydAB* bearing a vector were grown without IPTG. ZM4/pRL814 and Δ*cydAB/pRLnoxE* induced with IPTG were diluted to OD_600_ = 0.2 while Δ*cydAB/pRL814* and uninduced Δ*cydAB/pRLnoxE* were diluted to OD_600_=1.0**.** Oxygen partial pressure (pO_2_) was measured as described in Materials and Methods and in Figure S5. Each graph shows oxygen consumption in one well, representative for the indicated strain.

**
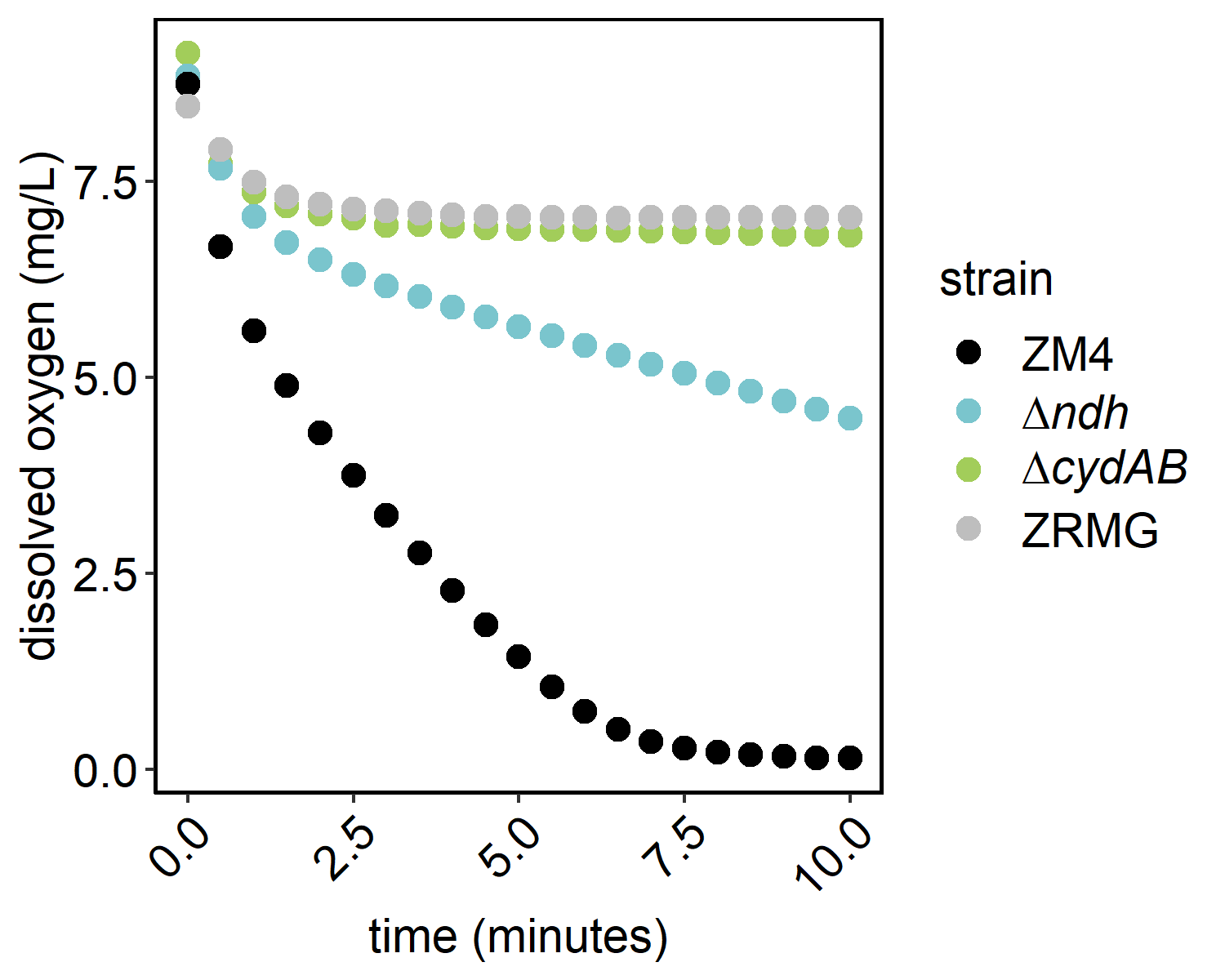
**

**Figure S9. Oxygen uptake in ZM4, Δ*ndh* and Δ*cydAB* measured with a manual probe.** Cultures were grown in ZRMG in anoxic conditions, overnight. They were diluted to OD_600_ = 1.0 in 40 ml of fresh aerobic ZRMG in 250 ml flasks and aerated by shaking for 30 minutes. The shaking was stopped and dissolved oxygen was measured immediately with an optical probe for 10 minutes. A representative graph is shown.
